# The effect of the Iranian health transformation plan on hospitalization rate: insights from an interrupted time series analysis

**DOI:** 10.1186/s12913-020-05186-6

**Published:** 2020-04-19

**Authors:** Siavash Beiranvand, Mandana Saki, Meysam Behzadifar, Ahad Bakhtiari, Masoud Behzadifar, Mohammad Keshvari, Nicola Luigi Bragazzi

**Affiliations:** 1 Social Determinants of Health Research Center, Lorestan University of Medical Sciences, Khorramabad, Iran; 2 Health Management and Economics Research Center, Iran University of Medical Sciences, Tehran, Iran; 3 Department of Health Management and Economics, School of Public Health, Tehran University of Medical Science, Tehran, Iran; 4 Vice Chancellor Treatment, Lorestan University of Medical Sciences, Khorramabad, Iran; 5 School of Public Health, Department of Health Sciences (DISSAL), University of Genoa, Genoa, Italy; 6 Laboratory for Industrial and Applied Mathematics (LIAM), Department of Mathematics and Statistics, York University, Toronto, Canada

**Keywords:** Health policy, Interrupted time series analysis, Health transformation plan, Hospitalization rate, Iran

## Abstract

**Background:**

Healthcare policy- and decision-makers make efforts to build and maintain high-performing and effective health systems, implementing effectiveness programs and health reforms. In May 2014, the Iranian Ministry of Health and Medical Education has launched a series of ambitious reforms, known as the Health Transformation Plan (HTP). This study aimed to determine the effect of the HTP on hospitalization rate in Iranian public hospitals affiliated to the Ministry of Health and Medical Education.

**Methods:**

This study was designed as a quasi-experimental, counterfactual study utilizing the interrupted time series analysis (ITSA), comparing the trend of hospitalization rate before and after the HTP implementation in 16 hospitals in the Lorestan province. Data was collected from March 2012 to February 2019.

**Results:**

In the first month of the HTP implementation, an increase of 2.627 [95% CI: 1.62–3.63] was noted (*P* < 0.001). Hospitalization rate increased by 0.68 [95% CI: 0.32–0.85] after the HTP implementation compared to the first month after the launch of the HTP (*P* < 0.001). After the HTP implementation, monthly hospitalization rate per 1000 persons significantly increased by 0.049 [95% CI: 0.023–0.076] (*P* < 0.001).

**Conclusions:**

The HTP implementation has resulted in an increased hospitalization rate. Health planners should continue to further improve this service. ITSA can play a role in evaluating the impact of a given health policy.

## Background

Healthcare policy- and decision-makers make serious efforts to build and maintain high-performing and effective health systems, which deliver high-quality services, while ensuring equity in access and sustainability. Improving health indicators represent their top priority. To reach such ambitious goals, they continuously strive to implement effectiveness programs and health reforms in their countries [1]. The health system seeks to provide more and better healthcare services, meeting with the different needs of the subjects, which are influenced and shaped by social, cultural, economic and political variables [2].

As health needs increase, the implementation of effective policies to promote equity in the health sector is a major concern for policy- and decision-makers [3]. Having a good level of health is one of the most important needs and fundamental rights of people. In part because of the increasing health challenges, the rapid progress of diagnosing and treating diseases, and the diversification of services in this sector, reforms of the health sector are inevitable and of paramount importance [4].

In May 2014, the Iranian Ministry of Health and Medical Education (MoHME) has launched a series of ambitious reforms, known as the Health Transformation Plan (HTP) [5, 6]. Nine main packages of healthcare services and provisions were considered as the core of the plan, including: i) reducing out-of-pocket expenditure, ii) increasing healthcare coverage, especially in remote, rural areas and recruiting physicians, healthcare workers and personnel in underserved areas, iii) providing specialist doctors in hospitals, iv) improving the quality and timing of patient visits, v) enhancing the quality of accommodation services, vi) promoting natural childbirth, by reducing the choice of cesarean section, vii) ensuring that diseases with long and expensive treatment are covered by adequate financial protection, viii) changing health tariffs to reduce informal payments and promote cost-effective interventions, and ix) building ambulance helicopter base centers [5]. This has enabled to provide health services to nearly 9–10 million people from marginalized areas in Iran [5].

HTP implementation guidelines state that “all hospitals affiliated with the MoHME are subject to HTP” and, as such, the effects of the HTP can be observed for the above-mentioned hospitals, while private hospitals are not included in the HTP. At the time of the HTP implementation, 570 hospitals affiliated to the MoHME and 337 private hospitals were active [5, 7, 8]. According to the MoHME policies, public hospitals in each province should cover the needs of women and children, surgeries, public referrals, and should ensure healthcare provisions for each type of hospital (general or specialized hospital, characterized by specialized equipment) and division [5–7].

The development and implementation of this project has increased public confidence in public healthcare systems as they represent the backbone of the public and community health system [8]. In particular, special attention has been paid to the delivery and provision of high-quality services in hospitals [9]. Hospitalized patients expect, indeed, to receive the most appropriate and high-quality services in hospitals due to their crucial role in the treatment of their diseases, to ensure their quick recovery and return to health and well-being [10].

Guaranteeing access to various health services by reducing patients queue, benefiting from specialist doctors, reducing medical costs and tariffs, and promoting hospitality ensure compliance to the process of disease treatment and management [11, 12]. After the approval of the HTP, health policy- and decision-makers in Iran have tried to provide a wide range of services in public hospitals and improve their health indicators [9, 13].

Moreover, since the implementation of the HTP, various studies have investigated its effect on the health system’s functions in Iran and have attempted to evaluate its various aspects [14, 15].

Conducting such studies can provide quantitative, updated, evidence-based information on the effects of the project of the implementation. This information is especially valuable for healthcare planners and executives, who are provided with an unbiased overview of the current status of the health system, which can be compared with the previous situation, assessing the effectiveness and efficiency of the plan and its expected benefits. This has also helped Iranian policy- and decision-makers to better plan and improve Iranian healthcare systems by reducing and solving the implementation issues of the HTP [7, 16, 17].

Given the importance of studies evaluating the effects of the HTP, the present study aimed to determine the impact of the HTP on hospitalization rate in Iranian hospitals affiliated to MoHME.

## Methods

Lorestan, with its 28,294 km^2^ and a population of 1,760,649 inhabitants (data referring to 2016), is one of the western provinces of Iran. It has 11 cities, the capital of which is Khorramabad, and nine cities have hospitals affiliated to the MoHME.

This study was designed as a quasi-experimental, counterfactual study and was analyzed and interpreted utilizing the interrupted time series analysis (ITSA). In this study, the effect of an intervention (namely, the implementation of the HTP) was assessed [18] by statistically comparing the trend of hospitalization rate before and after the HTP implementation in hospitals affiliated to the MoHME. The investigation was based on data collected monthly between March 2012 and February 2019 from 16 hospitals affiliated to the MoHME. in the Lorestan province.

The information of the hospitals affiliated to the MoHME in the Lorestan province was obtained through the Vice Chancellor of the Lorestan University of Medical Sciences. Each month hospitals sent the hospitalization rate to the Vice Chancellor. Demographic information was also collected through the National Statistics Center of Iran. There were no private hospitals in the Lorestan province before the HTP implementation, and two years after the HTP implementation, a private hospital started operating.

In the Lorestan province, there was a hospital affiliated to the Social Security Organization, which did not provide researchers with access to the hospital’s data, so this hospital was excluded from the present investigation. Khorramabad has the largest number of hospitals with respect to other provinces and, in many cases, patients are transferred from other provinces and cities’ hospitals to Khorramabad hospitals for treatment and management.

The following segmented regression model was built [19]:

$$ {\mathrm{Y}}_{\mathrm{t}}={\upbeta}_0+{\upbeta}_1\ast {\mathrm{t}\mathrm{ime}}_{\mathrm{t}}+{\upbeta}_2\ast {\mathrm{HTP}}_{\mathrm{t}}+{\upbeta}_3\ast \mathrm{after}\_{\mathrm{HTP}}_{\mathrm{t}} $$

β_0_ represents the initial hospitalization rate at the commencement of the study. Time_t_ is the temporal interval from baseline. β_**1**_ is the slope of the hospitalization rate before the HTP was launched. β_2_ is the slope of the hospitalization rate at the first month of the HTP implementation. β_3_ is the slope of the hospitalization rate after the HTP implementation as observed in the following months. Finally, after_HTPt is the time passed after the HTP implementation.

We used the Newey-West approach in our estimating analytical approach [20]. We also conducted several diagnostic and sensitivity assessments. The results of the Dickey-Fuller statistics suggested whether the residuals were stationary and normally distributed.. Ordinary least squares (OLS) regression model with a time series specification (an intercept and a trend term, a level and a trend change) was utilized in order to check for serially correlated errors by visualizing the residuals from the OLS regression and plotting the autocorrelation and partial autocorrelation (ACF/PACF) graphs [18].

Results were computed with their 95% confidence interval (CI) and *p*-values < 0.05 were considered as statistically significant. The open source R Ver 5.3.2 software was used for all data analyses.

## Results

Additional file 1 shows the results of the data analysis on hospitalization rate in the Lorestan province from March 2012 to February 2019. The observations of our study cover a span of 84 months. Specifically, the number of hospitalization rates observed by month and year is reported in Table 1.

**Table 1:** Hospitalization rate in each city in Lorestan providence

Year	Aleshtar	Aligodarz	Azna	Boroujerd	Dorud	Khorramabad	Kohdasht	Noorabad	Pol-Dokhtar	All
2012	1832	10,286	4032	18,703	8855	35,097	16,904	5546	3691	106,958
2013	1884	11,846	3486	22,970	9644	51,033	20,026	7180	3602	133,684
2014	3664	12,663	3497	22,829	10,531	57,822	20,701	6802	4627	145,150
2015	5933	13,737	5373	25,494	11,591	79,363	21,425	7938	4962	177,831
2016	5773	14,352	5201	26,612	11,383	71,376	23,852	7783	8178	176,526
2017	5196	17,288	4908	26,968	11,109	77,595	22,087	8254	7098	182,520
2018	4041	16,306	3135	27,757	10,904	9119	22,791	7373	7883	111,327
2019	510	2304	636	4236	1785	14,356	3242	1243	1377	31,708

According to the findings of the present investigation, the mean hospitalization rate per 1000 persons was 11.07 during the study period. More in detail, before the HTP implementation, the temporal trend was slightly decreasing with a monthly reduction by 0.018 [95% CI: 10.72–11.4], which, however, was not statistically significant (*P* = 0.11).

In the first month of the HTP implementation in the Lorestan province, an increase of 2.627 [95% CI: 1.62–3.63] was noted and achieved the significance threshold (*P* < 0.001).

Patients’ monthly hospitalization rate increased by 0.68 [95% CI: 0.32–0.85] after the HTP implementation compared to the first month after the launch of the HTP, which was still statistically significant (*P* < 0.001). After the HTP implementation, monthly hospitalization rate per 1000 persons significantly increased by 0.049 [95% CI: 0.023–0.076] (*P* < 0.001). Table 2 shows the various coefficients of the segmented regression model.

**Table 2:** Findings from the segmented regression model

Parameter	Coefficients	Newey-West Standard Errors	*P* Value	Lower of CI (95%)	Upper of CI (95%)
Intercept	11.070	0.171	0.000	10.729	11.410
Preintervention slope	−0.018	0.011	0.111	−0.040	0.004
Change in intercept	2.627	0.504	0.000	1.622	3.631
Change in slope	0.680	0.017	0.000	0.032	0.103
Post intervention linear trend	0.049	0.013	0.000	0.023	0.076
F (3, 80)	149.73				
Number of observations	84				
Prob > F	0.000				

**Fig. 1:**
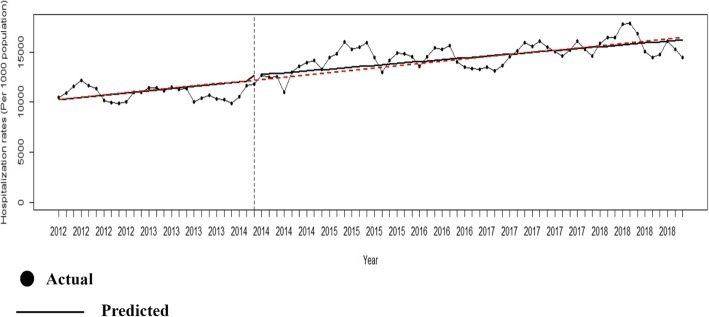
Temporal trend of the hospitalization rate in the Lorestan governmental, public hospitals before and after the Health Transformation Plan implementation

**Fig. 2:**
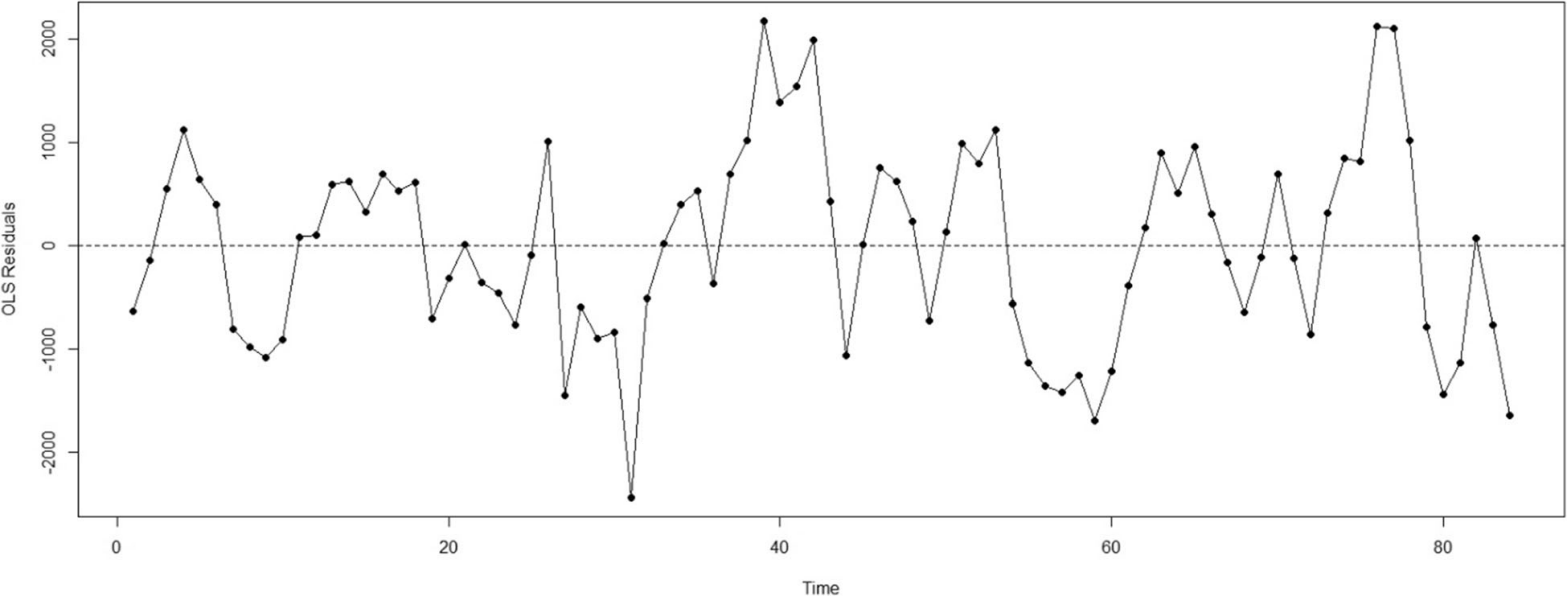
Result of residuals from the OLS regression of hospitalization rate

**Fig. 3:**
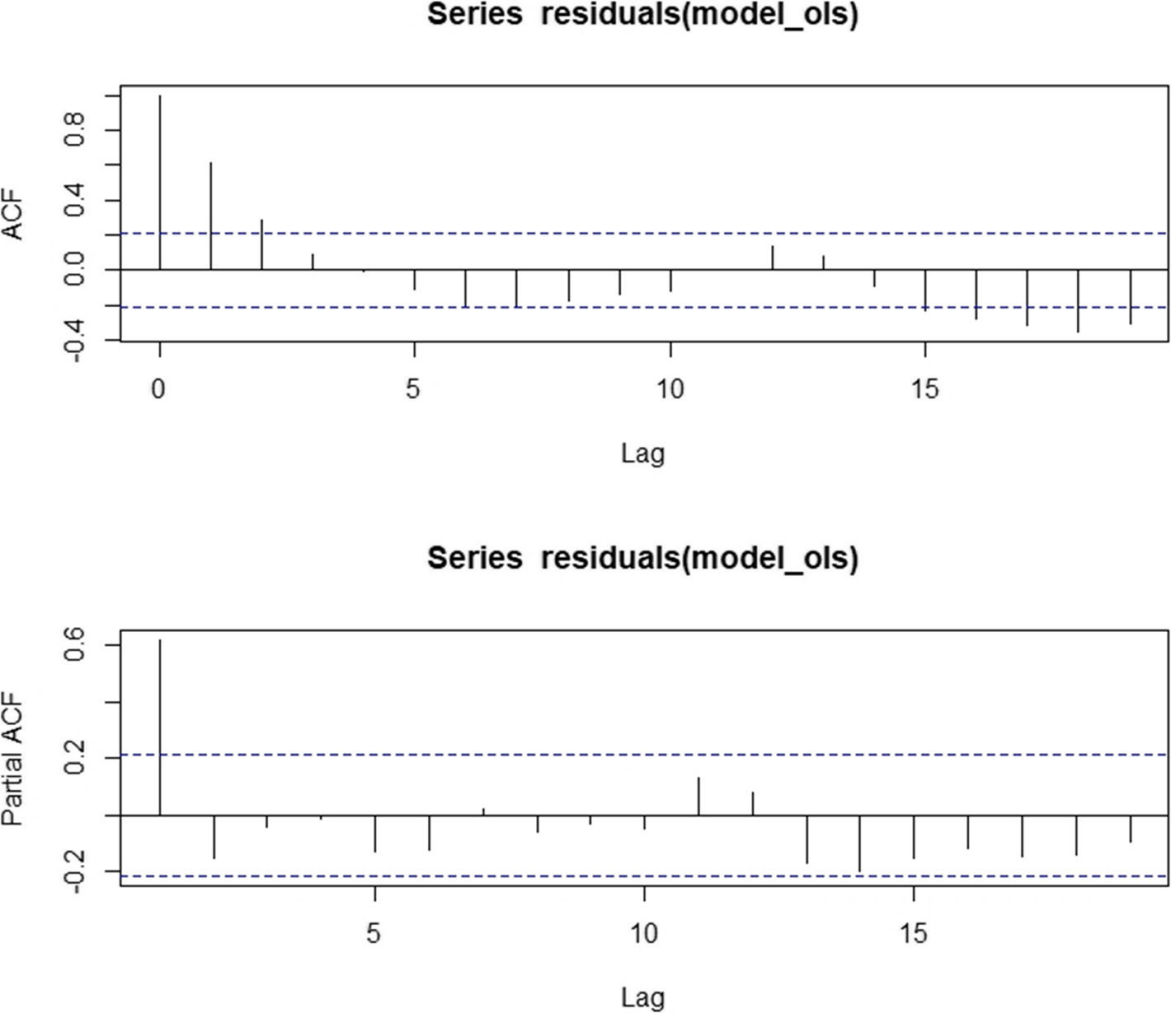
Autocorrelation and partial autocorrelation function of the of hospitalization rate

Figure 1 pictorially shows the temporal trend of the hospitalization rate for governmental, public hospitals in the Lorestan province before and after the HTP implementation. The OLS regression of hospitalization rate in hospitals affiliated to the MoHME is shown in Fig. 2. The ACF/PACF plots of the residuals are reported in Fig. 3.

Post-intervention linear trends after the HTP implementation in terms of changes in the hospitalization rate based on the New-West standard errors for different cities of the Lorestan province are listed in Table 3.

**Table 3:** Coefficients of the segmented regression model after the Health Transformation Plan implementation in different cities of the Lorestan province

City	Coefficients	Lower of CI (95%)	Upper of CI (95%)	Newey-West Standard Errors	F (3,80)	*P* Value
Aleshtar	−0.002	−0.005	0.001	0.030	21.79	0.000
Aligodarz	0.005	0.002	0.008	0.001	30.87	0.000
Azna	−0.001	−0.005	0.001	0.001	43.98	0.264
Boroujerd	0.005	0.002	0.008	0.001	53.05	0.000
Droud	−0.000	−0.000	0.000	0.000	6.36	0.846
Khorramabad	0.036	0.017	0.055	0.009	63.72	0.000
Kohdasht	0.000	−0.003	0.004	0.002	12.05	0.820
Noorabad	0.000	−0.001	0.002	0.001	7.80	0.851
Pol-Dokhtar	0.006	0.004	0.007	0.009	74.17	0.000

After the HTP implementation, hospitalization rate in Aligodarz, Boroujerd, Khorramabad, Kohdasht and Noorabad and Pol-Dokhtar cities increased, whereas the trend was declining in Aleshtar, Azna and Droud cities. Changes were statistically significant (*P* < 0.001) for all cities except for Azna, Droud, Kohdasht and Noorabad.

## Discussion

The present study was conducted in order to investigate the effects of the HTP implementation on the hospitalization rate in governmental, public hospitals of the Lorestan province, situated in western Iran.

The implementation of this plan has been one of the most important policies in recent decades in Iran and has received much political support and economic-financial funding [5]. Various studies have shown that users were satisfied with the services provided after the project was implemented [21]. The most common reason for patients’ satisfaction was the reduction of the out-of-pocket payments for hospitalized cases [22]. Although the percentage of out-of-pocket expenditure has declined, the costs of insurance agencies have increased by more than 70 % on average for each hospitalized patient [23].

In the first month after the HTP implementation, the hospitalization rate increased by 2.627, which is in line with the results of Karami Matin et al. [24] and of Saha et al. [25]. When discharged, recipients in public hospitals pay much less than before the HTP implementation. One of the most important goals of implementing the HTP was, indeed, to curb the out-of-pocket expenditure. One of the goals of universal health coverage is to reduce costs and increase equity in access to the health sector. Thus, lowering costs for patients has made people more interested in utilizing public hospital services and being more compliant to the process of disease treatment and management. Studies carried out in different parts of Iran have shown that out-of-pocket payments are in line with the target set by the HTP, and that the MoHME has been successful in reducing costs [5, 26].

Governmental, public economic support for patients can affect the process of hospitalization. Ensuring that costs are paid and that patients’ well-being is still ensured makes patients more likely to be hospitalized until they fully recover [27]. The findings of the present study showed that hospitalization rates increased monthly after the HTP. Following the implementation of HTP, health policy- and decision-makers have, indeed, made extensive efforts to improve services and have increased people trust and confidence towards public health services. Increasing the quality of services has made people more likely to utilize public hospitals and their services. In its turn, this increase in quality has been effective in changing the behavior of service recipients [28]. Universal health coverage cannot be achieved without increasing the quality of service [29]. The findings of the study by Maleki et al. in Iran showed that service providers are more likely to collaborate with hospitals that take the necessary steps to provide better quality services [30]. Providing access to nearly 9–10 million marginalized people who had no access to health services and performing basic health visits and assessments for them could be another important reason for the increase in hospitalizations [31].

Covering insurance and reducing out-of-pocket payments can increase referrals of marginalized subjects to hospitals, especially the public ones, and the utilization of public services is greater in the poorer income quintiles and more marginalized social groups [32].

One of the reasons that has led to the increased hospitalization rate was the presence of specialist doctors in public hospitals. The shortage of physicians and service providers in hospitals is one of the major problems in the Iranian health sector [33]. However, policy- and decision-makers have sought to increase access to medical care by increasing equity in access, ensuring high-quality services and provisions, with most patients benefiting from specialist physicians. In particular, access to health specialists in disadvantaged and less developed areas leads to improved health outcomes. The chronic shortage of medical staff and personnel in underdeveloped areas has hampered the provision of diagnostic and treatment services to disadvantaged and needy residents. Promoting health in less developed areas was one of the major benefits of implementing the HTP, which significantly increased the viability of medical staff in deprived areas. Due to the presence of specialist physicians, many patients were admitted to hospitals after being diagnosed, instead of being sent to major cities for referral. The presence of service providers is expected to further increase accessibility and sustainability in less developed areas [34].

In the years following the introduction and implementation of the HTP in Iran, the number of hospital beds has increased. With increasing funding from the health sector, policy- and decision-makers have been able to provide more services. Therefore, the capacity of admission and hospitalization in Iran, and especially in the Lorestan province, has also increased. This increase has made doctors more likely to hospitalize patients.

Cultural and social factors can influence treatment and management processes. One of the reasons for the increase in hospitalization rate in the Lorestan province is the cultural perspective [35]. In a US study, facilitated access to health services and their quality improvement reduced the rate of hospitalization, which is inconsistent with the findings of the present study. This difference can be due to cultural and social differences existing between the two countries and the organizational aspects of the two healthcare systems [36].

The findings of the present study showed that hospitalization rates were higher in some cities, which had larger populations and a growing number of specialist physicians. In addition, some cities were adjacent to other provinces in the country where patients are usually referred for services. Policy- and decision-makers have tried to make health services more and equally accessible in all cities, but some of them still have inadequate health infrastructure.

### Limitations

However, the present study is not without any limitations. Our data concerning the length of stay in hospital and the causes of hospitalization were incomplete. For this reason, we focused on hospitalization rate without performing any stratification analysis. Various variables can affect the process of hospitalization and, in our case, it was not possible to check and/or adjust for all these variables. Another limitation is given by the lack of data related to hospitals and healthcare settings of the private sector as comparisons. On the other hand, we focused only on public hospitals, give the aim of the HTP. Another shortcoming is given by the short-term perspective which has been employed in the present investigation. As such, further studies assessing long-term effects and impact of the HTP, are warranted in the field.

## Conclusion

The findings of the present study showed that, after the HTP implementation, facilitating access to health services and improving their quality have increased the rate of hospitalization in governmental, public hospitals of the Lorestan province. Health policy- and decision-makers should continue to make efforts to further improve healthcare services, given the people trust and confidence towards public hospitals, even though increase in hospitalization rates could be due also to unmet needs. ITSA can play an important role in evaluating the process and impact of a given health policy. The findings of these studies can be effective for policy- and decision-makers.

## Supplementary information

**Additional file 1.**

## Data Availability

Not applicable.

## Abbreviations

HTP: Health Transformation Plan
ITSA: Interrupted time series analysis
MoHME: Ministry of Health and Medical Education
OLS: Ordinary least squares
